# Exploring the effects of Hippo signaling pathway on rumen epithelial proliferation

**DOI:** 10.1186/s12917-024-04067-y

**Published:** 2024-05-10

**Authors:** Bin Yang, Zebang Xu, Yilang Qin, Ying Peng, Yang Luo, Jiakun Wang

**Affiliations:** 1 Key Laboratory of Molecular Animal Nutrition, Ministry of Education, Zhejiang University, Hangzhou, 310058 Zhejiang China; 2 School of Biological and Chemical Engineering, Zhejiang University of Science and Technology, Hangzhou, 310023 Zhejiang China; 3 Institute of Dairy Science, College of Animal Sciences, Zhejiang University, Hangzhou, 310058 Zhejiang China; 4 Hunan Institute of Animal and Veterinary Science, Changsha, 410131 Hunan China

**Keywords:** Rumen epithelium, Hippo signaling pathway, Proliferation, Development

## Abstract

**Background:**

The current understanding to the mechanism of rumen development is limited. We hypothesized that the Hippo signaling pathway controlled the proliferation of rumen epithelium (RE) during postnatal development. In the present study, we firstly tested the changes of the Hippo signaling pathway in the RE during an early growing period from d5 to d25, and then we expanded the time range to the whole preweaning period (d10-38) and one week post weaning (d45). An in vitro experiment was also carried out to verify the function of Hippo signaling pathway during RE cell proliferation.

**Results:**

In the RE of lambs from d5 to d25, the expression of baculoviral IAP repeat containing (*BIRC3*/*5*) was increased, while the expressions of large tumor suppressor kinase 2 (*LATS2*), TEA domain transcription factor 3 (*TEAD3*), axin 1 (*AXIN1*), and MYC proto-oncogene (*MYC*) were decreased with rumen growth. From d10 to d38, the RE expressions of *BIRC3*/*5* were increased, while the expressions of *LATS2* and *MYC* were decreased, which were similar with the changes in RE from d5 to d25. From d38 to d45, different changes were observed, with the expressions of *LATS1*/*2*, MOB kinase activator 1B (*MOB1B*), and *TEAD1* increased, while the expressions of *MST1* and *BIRC5* decreased. Correlation analysis showed that during the preweaning period, the RE expressions of *BIRC3*/*5* were positively correlated with rumen development variables, while *LAST2* was negatively correlated with rumen development variables. The in vitro experiment validated the changes of *LATS2* and *BIRC3*/*5* in the proliferating RE cells, which supported their roles in RE proliferation during preweaning period.

**Conclusions:**

Our results suggest that the *LATS2*-*YAP1*-*BIRC3*/*5* axis participates in the RE cell proliferation and promotes rumen growth during the preweaning period.

**Supplementary Information:**

The online version contains supplementary material available at 10.1186/s12917-024-04067-y.

## Background

Promoting the healthy growth of ruminants is important to meet the increasing demand of the world’s population for high-quality animal protein. Rumen is the unique organ of ruminants, which is a large fermentation chamber that constitutes 80% of the total stomach volume [1]. It digests forage to produce volatile fatty acids (VFAs), which provide the animal host with 70% of its daily energy [2]. While in the neonates, the rumen is undeveloped and only constitutes less than 25% of the total stomach volume [1]. Improving rumen development can enhance the slaughter performance and milk yield of dairy calves [3, 4]. However, the current understanding to the mechanism of rumen development is still limited, which remains a barrier to achieving such improvement.

Attempts have been made to uncover the pathways and endogenous factors involved in rumen growth. It was reported that the PI3K-Akt [5], IGF-1 [6], MAPK, Jak-STAT, Ras [7], Hippo, Wnt, and thyroid hormone signaling pathways [8], as well as PPARα, PPARδ, and pirinixic acid [9] might be involved in the proliferation of rumen epithelium (RE). While most of these results were generated by transcriptomics and needed further validation. Among these pathways, the Hippo signaling pathway is a negative master controller of organ size and tissue regeneration by limiting cell growth [10, 11]. In the mammals, the Hippo kinase cascade contains STe20-like kinases (*MST1*/*2*) and large tumor suppressor kinases (*LATS1*/*2*) [12]. The output of the LATS-mediated phosphorylation pathway is the Yes1-associated transcriptional regulator (*YAP1*) and the WW domain-containing transcription regulator 1 (*TAZ*), which participate in various biological processes [13]. The modulators of the Hippo kinase include Salvador family WW domain-containing protein 1 (*SAV1*) and MOB kinase activator 1A/B (*MOB1A*/*B*), which regulate the dimerization of LATS [14]. Changes in each Hippo kinase and its modulators can regulate cell proliferation, cell differentiation, and tissue regeneration under different conditions [15–17]. While it is not clear whether the Hippo signaling pathway controls the RE proliferation during postnatal development.

The cultured RE cell serves as a critical and high-value tool to reveal the key players in RE cell proliferation, inflammation, and metabolic function [18, 19]. By utilizing the in vitro RE cell model, the functions of G protein-coupled receptor 41 (*GPR41*) and the PIK3-AKT-mTOR pathway [20], period circadian regulator 2 (*PER2*) [18], and insulin-like growth factor-binding proteins (*IGFBP2*/*3*/*6*) [21] in RE cell proliferation have been confirmed. Thus, it provides us with an appropriate in vitro model to validate the relationship between the Hippo signaling pathway and RE cell proliferation. Resveratrol is a nutritional additive for ruminants that can modify rumen fermentation, decrease methane production, and promote animal health [22, 23]. Resveratrol is also a modulator of the Hippo signaling pathway. It can suppress the Hippo signaling pathway and activate YAP by inhibiting *MST1* in cardiomyocytes to ameliorate myocardial ischemia/reperfusion [24], or by inhibiting *LATS1*/*2* in bone marrow mesenchymal stem cells to reverse the impaired osteogenic differentiation [25]. Verteporfin is a disruptor of *YAP*/*TAZ*-*TEAD* mediated transcription that used to inhibit the effect of Hippo signaling pathway [26, 27]. Thus, the resveratrol and verteporfin could be proper tools for studying the function of Hippo signaling pathway in vitro.

We hypothesized that the Hippo kinases and the associated modulators played roles in RE cell proliferation from pre- to postweaning period. According to the change of rumen weight from d10 to d66 lambs in our previous study, d24 is the beginning for fast growth of rumen [28]. From d24 to d38, the weight of the rumen increased from 32.82 g to 158.74 g in a span of two weeks, resulting in a growth rate of 9.00 g/d. While before d24, the rumen is small and maintains a low growth rate of 1.46 g/d from d10 to d24 [28]. Thus, in the present study, we first tested the changes of genes related to the Hippo signaling pathway in the RE during a transition from a low growth rate state (d5) to a high growth rate state (d25). Then, the changes of Hippo signaling pathway in the RE from pre- (d10 and d38) to postweaning period (d45) and their relationships with rumen growth were also analyzed. At last, an in vitro experiment of RE cells treated with resveratrol and verteporfin was carried out to verify the function of Hippo signaling pathway during RE cell proliferation. We aimed to investigate whether the Hippo signaling pathway can regulate rumen growth during development.

## Results

### Transcriptomic expressions of Hippo signaling pathway related genes in the RE of d5-25 lambs

**Fig. 1:**
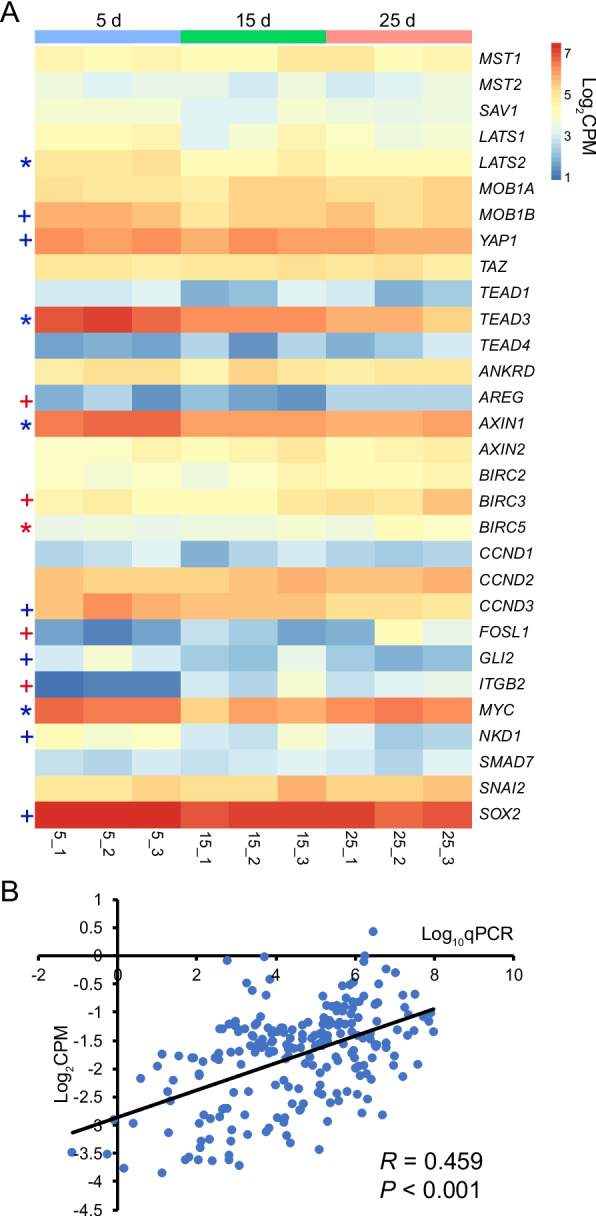
Expressions of the Hippo signaling pathway related genes in the rumen epithelium of lambs from d10 to 25. (**A**) Heatmap shows the transcriptomic expressions of the Hippo signaling pathway related genes. The significance of age effect is marked with * (represents *P* ≤ 0.05) and + (represents 0.05 < *P* ≤ 0.10). * and + in red and blue represent increase and decrease, respectively. (**B**) Correlation between the expression levels of genes related to the Hippo signaling pathway generated by RNA sequencing and quantitative PCR

From d5 to d25, for the Hippo kinases, the mRNA expression of *LATS2* was significantly decreased (*P* ≤ 0.05, Fig. 1A). For the downstream genes, the mRNA expression of baculoviral IAP repeat containing 5 (*BIRC5*) was significantly increased, while the TEA domain transcription factor 3 (*TEAD3*), axin 1 (*AXIN1*), and MYC proto-oncogene (*MYC*) were significantly decreased (*P* ≤ 0.05, Fig. 1A). Other genes did not show a significant change with increasing age (*P* > 0.05). While the amphiregulin (*AREG*), baculoviral IAP repeat containing 3 (*BIRC3*), FOS-like antigen 1 (*FOSL1*), and integrin subunit beta 2 (*ITGB2*) showed a tendency to increase, and *MOB1B*, *YAP1*, cyclin D3 (*CCND3*), GLI family zinc finger 2 (*GLI2*), naked cuticle homolog 1 (*NKD1*), and SRY-box transcription factor 2 (*SOX2*) showed a tendency to decrease from d5 to d25 (0.05 < *P* ≤ 0.10, Fig. 1A).

According to the qPCR analysis, only *AXIN1* showed a significant decrease from d5 to d25 (*P* ≤ 0.05, Table 1), which was consistent with the gene expression observed through RNA sequencing. The correlation between the gene expression data from RNA sequencing and qPCR showed a significant positive relationship (*P* < 0.001) with a coefficient of 0.459 (Fig. 1B). This suggests consistent results between the two methods.

**Table 1:** The relative gene expression of genes validated by quantitative PCR in the rumen epithelium of d5-d25 lambs

Gene	Day of age			SEM	*P*-value
	5	15	25		
*MST2*	0.21	0.03	0.04	0.037	0.113
*SAV1*	0.51	0.06	0.06	0.099	0.066
*LATS1* (E-2)	4.39	2.29	4.02	0.467	0.066
*LATS2* (E-2)	0.26	0.66	1.51	0.474	0.875
*MOB1A*	0.13	0.08	0.10	0.014	0.148
*MOB1B*	0.41	0.03	0.03	0.084	0.066
*YAP1*	0.99	0.06	0.02	0.290	0.733
*TAZ* (E-2)	2.26	2.12	1.54	0.378	0.733
*TEAD1*	0.29	0.02	0.03	0.088	0.202
*TEAD3*	0.11	0.03	0.03	0.020	0.061
*AREG* (E-3)	4.17	3.18	7.37	1.412	0.393
*AXIN1* (E-2)	2.92	0.99	0.27	0.489	0.027
*AXIN2* (E-3)	2.74	2.92	3.43	0.626	0.875
*BIRC2* (E-2)	3.87	2.03	2.86	0.423	0.288
*BIRC3* (E-2)	2.22	2.49	4.90	0.621	0.301
*BIRC5* (E-2)	2.68	2.16	3.51	0.582	0.301
*CCND2*	0.20	0.18	0.14	0.029	0.733
*CCND3*	0.12	0.03	0.04	0.018	0.061
*GLI2* (E-2)	1.01	1.10	0.04	0.322	0.066
*ITGB2* (E-4)	6.21	4.30	4.77	1.126	0.733
*MYC*	0.36	0.38	0.10	0.110	0.875
*NKD1* (E-2)	2.14	1.02	0.18	0.397	0.113
*SMAD7* (E-3)	0.72	1.25	0.48	0.202	0.393
*SNAI2* (E-2)	4.25	4.90	1.64	0.873	0.252
*SOX2*	0.07	0.11	0.04	0.021	0.393

### Hippo signaling pathway in the RE of lambs from pre- to postweaning period

**Fig. 2:**
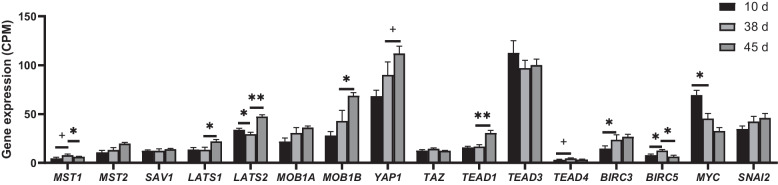
Expressions of the Hippo signaling pathway related genes in rumen epithelium of lambs from d10 to 45. ** represents *P* ≤ 0.01, * represents *P* ≤ 0.05, and + represents 0.05 < *P* ≤ 0.10

To analyze whether the changes in the Hippo signaling pathway and its related genes observed in d5-25 were consistent during other periods of rumen development, we further expanded the age range of the lambs from pre- (d10 and 38) to postweaning period (d45). Compared to d10, the mRNA expressions of *BIRC3*/*5* were significantly increased at d38, while the mRNA expressions of *LATS2* and *MYC* were significantly decreased at d38 (*P* ≤ 0.05, Fig. 2). Other genes did not show significant changes, while *MST1* and *TEAD4* exhibited increasing trends (0.05 < *P* ≤ 0.10, Fig. 2). Compared to d38, the mRNA expressions of *LATS1*/*2*, *MOB1B*, and *TEAD1* were significantly increased at d45, while the mRNA expressions of *MST1* and *BIRC5* were significantly decreased at d45 (*P* ≤ 0.05, Fig. 2). The *YAP1* showed an increasing trend from d38 to 45 (0.05 < *P* ≤ 0.10, Fig. 2).

### Relationship between Hippo signaling pathway and rumen growth

**Fig. 3:**
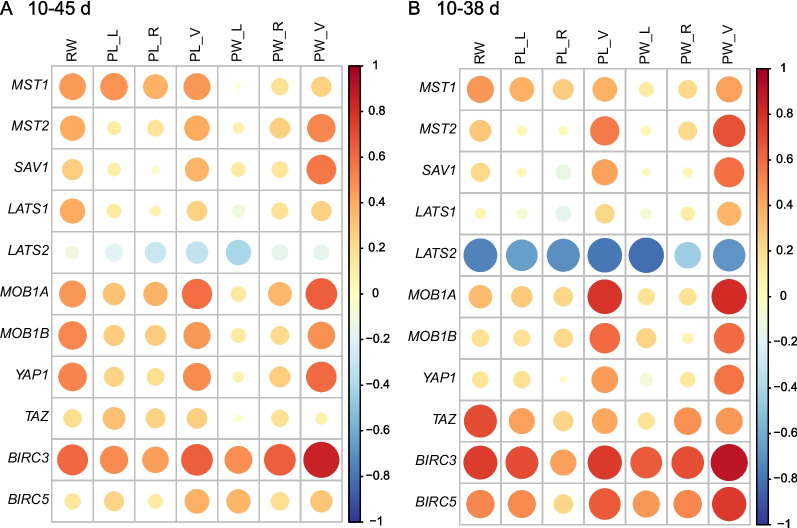
Spearman’s correlation between the Hippo signaling pathway related genes and rumen development variables. The color and dot size represent the correlation coefficient. Red represents a positive correlation, and blue represents a negative correlation; a larger dot size represents a stronger correlation, and a smaller dot size represents a weaker correlation. RW, rumen weight; PL, rumen papillae length; PW, rumen papillae width; L, left side; R, right side; V, ventral sac

From d10 to d45, positive correlations were observed between *SAV1* and ventral sac papillae width, between *MOB1A* and ventral sac papillae length and width, between *MOB1B* and rumen weight, between *YAP1* and ventral sac papillae width, and between *BIRC3* and rumen weight, ventral sac papillae length and width, and right-side papillae width (*P* ≤ 0.05, Fig. 3A). When we focused solely on the preweaning period from d10 to d38, we observed positive correlations between *MST2* and ventral sac papillae width, between *MOB1B* and ventral sac papillae length and width, between *TAZ* and rumen weight, between *BIRC3* and rumen weight, left-side papillae length, ventral sac papillae length and width, and between *BIRC5* and ventral sac papillae width (*P* ≤ 0.05, Fig. 3B). Negative correlations were observed between *LATS2* and rumen weight, papillae length of left, right, and ventral sac, and papillae width of left and ventral sac (*P* ≤ 0.05, Fig. 3B).

### Validation of the changes of Hippo signaling pathway related genes in proliferating RE cells

**Fig. 4:**
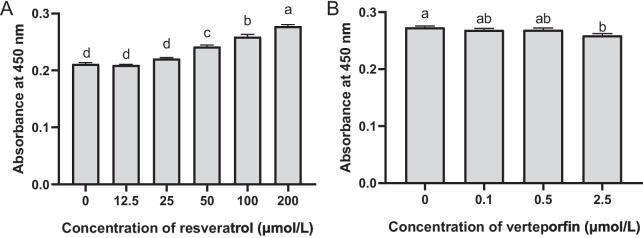
Rumen epithelial (RE) cell proliferation treated with resveratrol and verteporfin. (**A**) RE cell proliferation treated with different concentrations of resveratrol. (**B**) RE cell proliferation treated with different concentrations of verteporfin. Different lowercase letters (a-d) on the bar chart indicate significant differences (*P* ≤ 0.05)

**Fig. 5:**
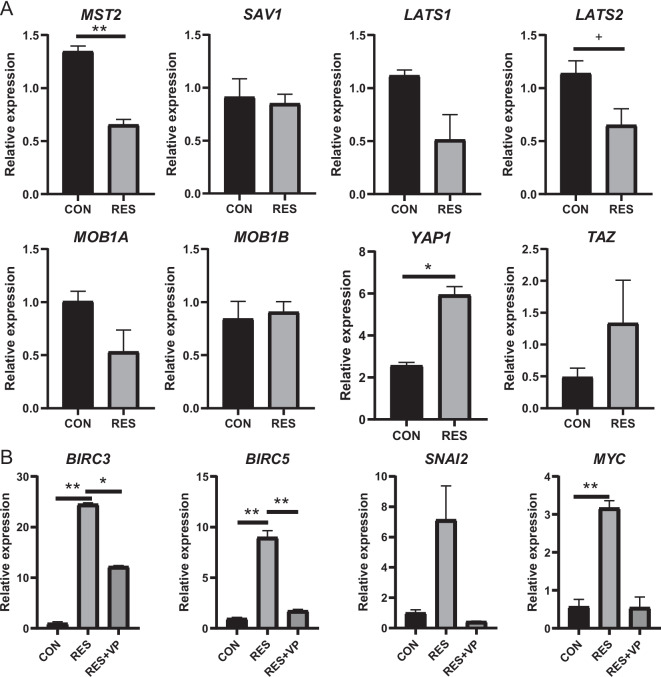
Expressions of the Hippo signaling pathway related genes in proliferating rumen epithelial (RE) cells. (**A**) Expressions of the Hippo signaling pathway members in the RE cells treated with resveratrol (RES) or not (CON). (**B**) Expressions of the Hippo signaling pathway downstream genes in the RE cells treated with resveratrol (RES), resveratrol and verteporfin (RES + VP), or not (CON). ** represents *P* ≤ 0.01, * represents *P* ≤ 0.05, and + represents 0.05 < *P* ≤ 0.10

By treating with different concentration of resveratrol, we identified that 12.5 and 25 μmol/L resveratrol had no impact on RE cell proliferation, while 50, 100, and 200 μmol/L resveratrol showed significant stimulation to cell proliferation (*P* ≤ 0.05, Fig. 4A). The cell proliferation significantly increased with the increasing concentration of resveratrol from 50 to 200 μmol/L (*P* ≤ 0.05, Fig. 4A). Then, we measured the mRNA expressions of genes related to the Hippo signaling pathway in RE cells treated with 200 μmol/L resveratrol (RES) or not (CON) (*P* ≤ 0.05, Fig. 5A). We observed that the mRNA expressions of *MST2* and *LATS2* were significantly decreased, while the expression of *YAP1* was significantly increased in resveratrol-treated RE cells compared to the CON (*P* ≤ 0.05, Fig. 5A). This suggested that the Hippo signaling pathway in RE cells was suppressed by resveratrol. For the downstream genes, the mRNA expressions of *BIRC3*/*5* and *MYC* were significantly increased in the resveratrol-treated RE cells (*P* ≤ 0.05, Fig. 5B).

We observed that RE cell proliferation was not affected when treated with 0.1 or 0.5 μmol/L verteporfin (Fig. 4B). While when the concentration of verteporfin increased to 2.5 μmol/L, the RE cell proliferation was significantly inhibited (*P* ≤ 0.05, Fig. 4B). By treating with 200 μmol/L resveratrol plus 2.5 μmol/L verteporfin, the mRNA expressions of *BIRC3*/*5* and *MYC* were significantly decreased in the RE cells compared to RES (*P* ≤ 0.05, Fig. 5B). These findings suggest that inhibiting the Hippo signaling pathway with resveratrol can stimulate RE cell proliferation, while disrupting the Hippo signaling pathway with verteporfin can inhibit RE cell proliferation.

## Discussion

It has been certified that the Hippo signaling pathway plays critical roles in the pregnancy recognition and establishment [29], as well as embryo development of ruminants [30, 31]. While it is not clear whether it participates in the gastrointestinal tract development of ruminants. The present study expanded knowledge of the Hippo signaling pathway in the proliferation of RE during rumen development, through both in vivo and in vitro experiments.

From the pre- (d5) to postweaning period (d45), the Hippo signaling pathway was expressed in the RE tissue, with some genes fluctuating during this period. These findings suggest that this pathway may play a role in the postnatal development of RE. During the preweaning period, the gene expression data from both d5-25 lambs and d10-38 lambs showed similar changes, including decreased expressions of *LATS2* and *MYC*, and increased expressions of *BIRC3*/*5*. The changes in *LATS2* and *BIRC3*/*5* were consistent in cultured RE cells treated with resveratrol. The decreased *LATS2* reduced the direct phosphorylation to *YAP1*, thus increased the nuclear localization of *YAP1* [32, 33]. With *YAP1* activation, *BIRC3*/*5* and *MYC* are the downstream targets of *YAP1* under specific conditions, such as in cystic kidney epithelium and colorectal cancer cells [34–36]. The *BIRC3*/*5* are upregulated in cancer tissues, leading to the promotion of cell proliferation [37, 38]. These results suggest that the Hippo signaling pathway regulates RE cell proliferation through decreased *LATS2* and increased *BIRC3*/*5* during the preweaning period. The significant correlation between rumen growth variables and the expressions of *LATS2* and *BIRC3*/*5* also supports the role of the *LATS2*-*BIRC3*/*5* axis in rumen growth. *MYC* plays multiple roles in cell growth and immune response by controlling the transcription of genes involved in proliferation, replication, apoptosis, differentiation, and metabolism [39–41]. Decreased *MYC* expression was reported in the submandibular gland of goats from 1 to 12 months of age to participate in the development of immune function [42]. Besides the Hippo signaling pathway, *MYC* is regulated by multiple signaling pathways such as Wnt, RTK, Notch, and TGF-β. Thus, the decreased *MYC* expression in RE during the preweaning period might be due to the regulation of different signaling pathways and involvement in multiple functions. The opposite change of *MYC* expression between growing RE tissue and proliferating RE cultured cells suggests that the in vitro model has limitations in accurately reflecting the complex in vivo changes.

Weaning stress is a significant challenge during ruminant growth, characterized by various physiological responses, including reduced feed intake, body weight loss, and diarrhea [43, 44]. Weaning stress halted the fast growth of the rumen [28]. During this period (from d38 to d45), the increased expressions of *LATS1*/*2* and *MOB1B* directly inhibited the activation of *YAP1* [32, 33], and then led to the decreased expression of *BIRC5*. The changes in *LATS2* and *BIRC5* after weaning also supported their roles in promoting rumen growth. The decreased expression of *MST1* contradicted with the increased expression of *LATS1*/*2*. Despite for the *MST1*-*LATS1*/*2*-*YAP1* axis, *MST1* can activate *Nur77* to regulate embryo-epithelium interaction [45], inhibit *Nrf2* to promote nasal epithelium inflammation [46], and associate with synaptotagmin-like protein 1 (*JFC1*) to regulate neutrophil transmigration through the vascular basement membrane [47]. Since weaning stress involves complex physical responses, the decreased expression of *MST1* might be related to other changes in RE rather than cell proliferation. While further study is needed to confirm the role of *MST1*. When focusing on both the pre- and postweaning periods, the expressions of *LATS2* and *BIRC5* were not correlated with rumen growth, which might be due to the complex regulatory networks in the RE during the weaning transition [9].

## Conclusion

In summary, our work expands the role of the Hippo signaling pathway in ruminants. The results suggest that the *LATS2*-*YAP1*-*BIRC3*/*5* axis participates in the RE cell proliferation and promotes rumen growth during the preweaning period. During the weaning transition, *LATS2* is increased to inhibit RE cell proliferation by decreasing *BIRC5*. While we failed to find specific antibodies for detecting the lamb LATS1/2 and YAP1 protein, the expression levels of these proteins were not validated in the present study. On the other hand, *MST1* of the Hippo signaling pathway might be involved in the regulation during weaning stress. Further studies investigating the function of *MST1* during the weaning transition should be conducted.

## Methods

### Rumen epithelial sample collection from d5-25 lambs

The RE samples used for the present study were collected from a previous animal experiment [48]. In brief, three healthy male Hu lambs at the ages of d5, d15, and d25 were selected from a commercial sheep farm and sacrificed for RE samples. The lambs were group raised with their mothers in wooden pens with a slotted floor. All lambs were raised on ewe’s milk. From d10 of age, lambs were supplemented with ad libitum starter pellets (49.73% corn, 26.82% soybean meal, 20.23% wheat bran, 0.64% NaCl, 1.58% calcium hydrogen phosphate, and 1.00% premix containing Fe, Zn, Cu, Mn, Co, I, Se, VA, VD and VE, dry matter). Before slaughter, each lamb was received an intramuscular injection of Lumianning (0.001 mL/ kg body weight; Hua Mu, Changchun, China) to minimize suffering during sacrifice [49]. After complete loss of consciousness, as indicated by lying down with tongue extension and salivation, the lambs were sacrificed for samples by exsanguination. After sacrifice, the RE tissue was isolated from ventral sac of the rumen wall and cut into three pieces with 1.5 × 1.5 cm^2^ each. Then, the RE samples were rinsed in pre-cooled sterilized PBS (4 °C, pH = 6.8), and snap-frozen in the liquid nitrogen until they were stored at − 80 °C for long-term preservation.

### Transcriptomic gene expressions in the rumen epithelium of d5-25 lambs

The RNA sequencing data of the RE tissue samples from d5, d15, and d25 lambs in this study were previously generated and can be accessed at the Gene Expression Omnibus (https://www.ncbi.nlm.nih.gov/geo/) with the accession numbers GSE227043 and GSE200295. The normalized mRNA expressions were calculated as counts per million reads (CPM). The expressions of the Hippo kinases, their associated modulators, and downstream genes, including *MST1*/*2*, *SAV1*, *LATS1*/*2*, *MOB1A*/*B*, *YAP1*, *TAZ*, *TEAD1*/*3*/*4*, ankyrin repeat domain 29 (*ANKRD*), *AREG*, *AXIN1*/*2*, *BIRC2*/*3*/*5*, *CCND1*-*3*, *FOSL1*, *GLI2*, *ITGB2*, *MYC*, *NKD1*, SMAD family member 7 (*SMAD7*), snail family transcriptional repressor 2 (*SNAI2*), and *SOX2*, were obtained from the RNA sequencing data.

### Quantitative PCR analysis

For quantitative PCR (qPCR) analysis, total RNA was extracted from the RE samples using a total RNA extraction kit (Aidllab, Beijing, China) following the instructions. The concentration and purity of the extracted RNA was measured with a Nanodrop 2000 (Thermo Scientific, Wilmington, USA). A ReverTra Ace qPCR RT Kit (Toyobo, Osaka, Japan) was used to perform the reverse transcription. For each reaction, 1 μg total RNA were used in 20 μL total reaction volume according to the instruction. Primers used in the present study were designed using the Primer-BLAST tool in the Basic Local Alignment Search Tool (BLAST) of the National Center for Biotechnology Information (NCBI) (Table 2). The amplification products were sequenced and searched using BLAST to validate the specificity of these primers. The qPCR was performed with a 20 μl reaction volume using FastStart Universal SYBR Green Master (Roche, Basel, Switzerland) on the ABI 7500 Real-Time PCR system (Applied Biosystems Inc., Foster City, CA, USA). The relative mRNA expression levels were normalized to the expression of *GAPDH* [50] using 2^−(Ct of target genes−Ct of*GAPDH*)^.

**Table 2:** Information of the primers used for gene expression validation

Target genes	Primer sequence, from 5' to 3'	Source	Product size (bp)
*MST2*	F: TGTGTGGCTGACATCTGGTC R: GTCCAGCTCATCCTCATCCG	XM_027973186.1	369
*SAV1*	F: CTTCCTCCAGGATGGGAACG R: TGCAGGTACCAGAAGGGACT	XM_004010517.4	183
*LATS1*	F: CTTGGATACCACAGCCCGTT R: TGGTGTAGCAGATGCTTGGG	NM_001306113.1	130
*LATS2*	F: GCTCCCCTTTGCTAACGAGT R: GAACGATCTGCTCCTTGCCT	XM_015098193.2	200
*MOB1A*	F: GCACTGAAGCAAGCTGTCCA R: AGGTGCCAACTCACGCCTAT	XM_004006087.4	374
*MOB1B*	F: GCTTCTTGTTTGGGAGTCGC R: CCAACTGGTCCTGAACCCAA	XM_015096546.2	375
*YAP1*	F: GAGATCCCTGACGATGTGCC R: TCATGGCAAAACGAGGGTCA	XM_015100723.2	313
*TAZ*	F: TCGCCTGATCGCTGAATGTC R: CGCATCTCCACTGCTGACTT	XM_004022254.4	198
*TEAD1*	F: CAGTCACCTGCTCCACCAAA R: CCCCTGCATGGTGAGGTTTA	XM_027979380.1	437
*TEAD3*	F: GGACATCAAGCCCTTTGCAC R: GGGGCCCTTTCTCATAGAGC	XM_027958367.1	344
*AREG*	F: GCACATTTTTAGAGCAACTGGA R: GATAAATCACTGTCGACCATGC	XM_012180164.3	115
*AXIN1*	F: GAGAGTTCAGGTGTGGACCG R: GCGGACTTCCTTTGGCATTC	XM_027961205.1	278
*AXIN2*	F: TTGAGAAACGGGACCACTCG R: ATCCATCGACAGGACCTCCA	XM_027973983.1	353
*BIRC2*	F: CTCTCTTTCAACAGTTGACGTG R: AAGATGTTGGCAGCACTATTTC	XM_012095319.3	172
*BIRC3*	F: TCACAGTGATGATGTGAAATGC R: GGCTTGAACTCGACTAATGAAC	XM_012095320.3	154
*BIRC5*	F: ACCGCGTCTCCACGTTTAAG R: AAACACTGAGCCAAGTCGGG	XM_004013098.3	124
*CCND2*	F: GCTGAGAAGTTGTGCATTTACA R: GATGTGTTCGATGAAGTCATGG	NM_001127290.1	132
*CCND3*	F: CGCGCCTCTTACTTCCAGTG R: GGGAGACAGAATGGTCGGTG	NM_001127289.1	280
*GLI2*	F: AAGGCAGGGATGCAGAACTC R: GCAAGGGATGTCAGAGGCTT	XM_027964829.2	303
*ITGB2*	F: GATCAACGTCCCGATCACCT R: CACTCGCAGTTCTTCCCGAT	NM_001009485.1	237
*MYC*	F: CAATGAAAAAGCCCCCAAAGTA R: TTTGAGTTTCAACTGTTCTCGC	NM_001009426.1	133
*NKD1*	F: TGGACCTTCACCCTGTACGA R: AGGACGATGCCCTTTTTGCT	XM_027977825.1	188
*SMAD7*	F: ACCGTGTAAATGGGGAGCAG R: AATGTCCGAGAAGGGGCAAG	XM_012103894.3	269
*SNAI2*	F: TGATTATCTCCCCGTGTCTCTA R: CTTTCTTCTTCGTCGCTAATGG	NM_001126342.1	256
*SOX2*	F: GTCCTATTCTCAGCAGGGCA R: CTGGGACATGTGAAGTCTGCT	NM_001318074.1	214
*GAPDH*	F: GGGTCATCATCTCTGCACCT R: GGTCATAAGTCCCTCCACGA	Wang et al., 2009	176

### Transcriptomic gene expression and rumen development variables in the rumen epithelium of d10-45 lambs

The normalized mRNA expressions of the Hippo signaling pathway members and the downstream *TEAD1*/*3*/*4*, *BIRC3*/*5*, *MYC*, and *SNAI2* in the RE of pre- (d10 and d38) and postweaning (d45) lambs were obtained at Sequence Read Archive with the access number PRJNA540396 [51]. The corresponding rumen development parameters including rumen weight, rumen papillae length and width from the ventral sac, right side, and left side were obtained from our previous study [28].

### Isolation and culture of primary rumen epithelial cells

The RE tissues were obtained from newborn male Hu lambs immediately after they were sacrificed and then transported to the laboratory in ice-cold DMEM. The isolation and culture method of primary RE cells was performed according to Klotz et al. [52] and Yang et al. [53] with some modifications. In brief, the RE tissues were cut into pieces (1 cm^2^) and washed with ice-cold D-Hank’s balanced salt solution containing 500 U/ml penicillin, 500 μg/ml streptomycin, 100 μg/ml gentamycin, and 5 μg/ml amphotericin B for several times until the solution was free of any contaminants. Then, the tissues were pre-digested with a 0.25% trypsin–EDTA solution (Solarbio, Beijing, China) at 37 °C in a shaking warm-air bath for 30 min. Afterward, the digestion solution was discarded to remove the cornified cells. Subsequently, the tissues were digested with a 0.25% trypsin–EDTA solution for 1.5 to 2 h. Every 10 min, the digestion solution was harvested and replaced with fresh solution. The FBS (Gibco, USA) was used to stop the trypsinization process after cell harvest. The harvested solution was centrifuged at 300 × g for 5 min at 4 °C, and the digested solution was removed from the cell pellets. The cells were suspended in DMEM and filtered using a 150-μm nylon mesh. Cell pellets were resuspended in DMEM containing 2% FBS, 50 U/ml penicillin, 50 μg/ml streptomycin, and 1% mixed additive (including 25 ng/ml epidermal growth factor, 100 ng/ml hydrocortisone, 10 μg/ml insulin, 5 μg/ml transferrin, 87 ng/ml cholera toxin, and 1.3 × 10^–2^ ng/ml triiodothyronine). The cells were then cultured at 37 °C with 5% CO_2_. After incubating for 1 h, the unattached cells were removed by discarding the supernatant and replacing it with fresh medium.

### In vitrorumen epithelial cell experiment and cell proliferation analysis

To determine the effects of resveratrol on RE cell proliferation, resveratrol (Shanghai yuanye Bio-Technology Co., Ltd, China) was diluted with DMSO to different concentrations, including 0, 12.5, 25, 50, 100, and 200 µmol/L. To screen the concentration of verteporfin for disrupting the Hippo signaling pathway, verteporfin (TOPSCIENCE, Shanghai, China) was diluted with DMSO to achieve concentrations of 0, 0.1, 0.5, and 2.5 µmol/L. The final concentration of DMSO was less than 0.2% (v/v), which had no effect on RE cell proliferation (Additional file 1). The RE cells were cultured in 96-well plates with 2,000 cells per well. Cells were treated with different concentrations of resveratrol/verteporfin for 24 h. Cell proliferation assays were conducted using the Cell Counting Kit-8 (Beyotime, Shanghai, China). The cells were counted in tuplicate wells, and the growth curves of the mean absorbance at 450 nm were plotted. The concentration of resveratrol for promoting RE cell proliferation and the concentration of verteporfin for inhibiting RE cell proliferation were selected.

The following cell experiment consisted three groups as follows: the control group (CON) that treated with 0.2% DMSO; the resveratrol group (RES) that treated with the selected concentration of resveratrol; and the resveratrol plus verteporfin group (RES + VP) that treated with the selected concentrations of resveratrol and verteporfin. Cells were seeded in 6-well plates with 2,000 cells/100 µL. The cell experiment was performed in triplicate wells. After being treated for 24 h, the cells were harvested and stored at − 80 °C for gene expression analysis.

### Statistical analysis

Data analysis was performed using SPSS 20.0 (SPSS, Inc., Chicago, IL, United States). Gene expressions obtained from transcriptomic data and qPCR analysis were analyzed using the Kruskal–Wallis signed rank test. A significant change was observed with *P*-value ≤ 0.05, and a trend was observed with 0.05 < *P*-value ≤ 0.10. The correlation between gene expressions to rumen development variables were performed using Spearman’s rank correlation. The results were plotted using R software (version 3.3.0) with the package “corrplot” package.

### Supplementary Information

Additional File 1: Effects of DMSO on rumen epithelial proliferation.

## Data Availability

The datasets used and/or analyzed during the current study are available from the corresponding author on reasonable request.

## Abbreviations

RE: Rumen epithelium
*MST1*/*2*: STe20-like kinase 1/2
*LATS1*/*2*: Large tumor suppressor kinase 1/2
*YAP1*: Yes1-associated transcriptional regulator
*SAV1*: Salvador family WW domain-containing protein 1
*MOB1A*/*B*: MOB kinase activator 1A/B
*BIRC1*/*3*/*5*: Baculoviral IAP repeat containing 1/3/5
*TEAD1*/*3*/*4*: TEA domain transcription factor 1/3/4
*AXIN1*: Axin 1
*MYC*: MYC proto-oncogene
*AREG*: Amphiregulin
*FOSL1*: FOS-like antigen 1
*ITGB2*: Integrin subunit beta 2
*CCND3*: Cyclin D 3
*GLI2*: GLI family zinc finger 2
*NKD1*: Naked cuticle homolog 1
*SOX2*: SRY-box transcription factor 2
