# Supplementary figures and images for: Exploring the effects of Hippo signaling pathway on rumen epithelial proliferation
Source: BMC Vet Res. 2024 May 10;20:186. doi: 10.1186/s12917-024-04067-y (PMC11084078; doi:10.1186/s12917-024-04067-y)

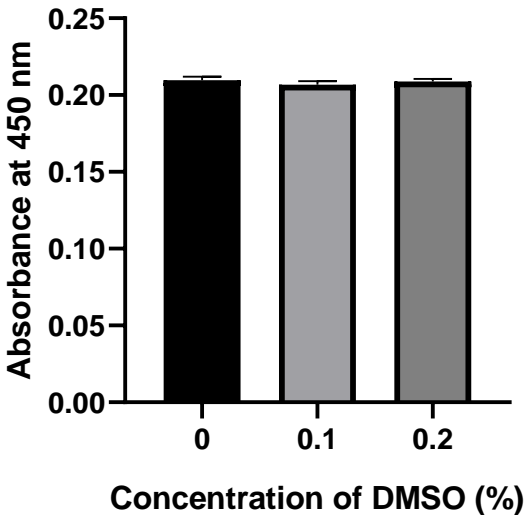

Supplement: Supplementary file 1 — Additional File 1: Effects of DMSO on rumen epithelial proliferation. [file 12917_2024_4067_MOESM1_ESM.pdf]
